# Proteoform identification and quantification based on alignment graphs

**DOI:** 10.1093/bioinformatics/btaf007

**Published:** 2025-01-09

**Authors:** Zhaohui Zhan, Lusheng Wang

**Affiliations:** Department of Engineering, Shenzhen MSU-BIT University, Shenzhen, 518172, China; Department of Computer Science, City University of Hong Kong, Hong Kong, 999077, China; Department of Computer Science, City University of Hong Kong, Hong Kong, 999077, China; City University of Hong Kong Shenzhen Research Institution, 518057, China

## Abstract

**Motivation:**

Proteoforms are the different forms of a proteins generated from the genome with various sequence variations, splice isoforms, and post-translational modifications. Proteoforms regulate protein structures and functions. A single protein can have multiple proteoforms due to different modification sites. Proteoform identification is to find proteoforms of a given protein that best fits the input spectrum. Proteoform quantification is to find the corresponding abundances of different proteoforms for a specific protein.

**Results:**

We proposed algorithms for proteoform identification and quantification based on the top-down tandem mass spectrum. In the combination alignments of the HomMTM spectrum and the reference protein, we need to give a correction of the mass for each matched peak within the pre-defined error range. After the correction, we impose that the mass between *any* two (not necessarily consecutive) matched nodes in the protein is identical to that of the corresponding two matched peaks in the HomMTM spectrum. We design a back-tracking graph to store such kind of information and find a combinatorial path (*k* paths) with the minimum sum of peak intensity error in this back-tracking graph. The obtained alignment can also show the relative abundance of these proteoforms (paths). Our experimental results demonstrate the algorithm’s capability to identify and quantify proteoform combinations encompassing a greater number of peaks. This advancement holds promise for enhancing the accuracy and comprehensiveness of proteoform quantification, addressing a crucial need in the field of top-down MS-based proteomics.

**Availability and implementation:**

The software package are available at https://github.com/Zeirdo/TopMGQuant.

## 1 Introduction

In top-down MS, multiplexed tandem mass (MTM) spectra are ubiquitous. An MTM spectrum arises when two or more proteoforms are analyzed using tandem MS, where these proteoforms remain indistinguishable within the spectrum and can not be well-separated by separation methods due to their similar masses (DiMaggio *et al.* 2009). The presence of the MTM spectra is not only due to the intrinsic complexity of the proteome, where a single gene can give rise to numerous proteoforms through processes such as alternative splicing, PTMs, and allelic variations but also due to limitations inherent to biological instrumentation (Zhu and Liu 2018). The high-throughput MS, while advantageous for proteomic research, often results in the co-ionization and co-fragmentation of proteoforms. This will also lead to MTM spectra where peaks corresponding to different proteoforms may overlap (Wang *et al.* 2010).

However, the study of these MTM spectra is critically important in top-down proteomic analysis (Wang *et al.* 2014). The ability to identify distinct proteoforms in an MTM spectrum and to accurately measure their relative abundances is crucial for a comprehensive understanding of the proteome’s functional landscape (Zhu and Liu 2018). This task is challenging due to the spectral overlap of co-eluting proteoforms, which can mask the presence of less abundant species and confound quantitative assessments. Precise identification and quantification are essential not only for elucidating the biological roles of individual proteoforms but also for identifying potential biomarkers and understanding the molecular underpinnings of diseases (Schaffer *et al.* 2019). The accurate representation of proteoform abundance is also critical for comparative studies, such as those investigating differential protein expression under various physiological conditions or in response to therapeutic interventions (Cesnik *et al.* 2018). Therefore, the development of methodologies that can effectively identify and quantify proteoforms from complex MTM spectra is a critical step toward top-down MS in proteomics research.

MTM spectra can be categorized into two types: heterogeneous MTM (HetMTM) spectra and homogeneous MTM (HomMTM) spectra. HetMTM spectra originate from the proteoforms of two or more different proteins, whereas HomMTM spectra are derived from proteoforms of a single protein that exhibit variations in their PTM patterns (Zhu and Liu 2018). The challenge with HomMTM spectra lies in the precise identification for the differences between the proteoforms and quantification for the relative abundance, which requires a detailed understanding of the potential modifications and their mass shifts (Kafader *et al.* 2020). Here, our research is based on HomMTM spectra. The task for HomMTM spectra analysis is multifaceted. Firstly, it involves identifying the original protein from which the proteoforms are arisen. This step is crucial as it is the guarantee for further characterization of the proteoforms and fundamental interpretation of the subsequent variations observed in the proteoforms. Once the original protein is identified, the next challenge is to determine the specific variant forms of each proteoform present in the HomMTM spectrum. In addition to identifying the proteoforms and their modifications, another critical aspect of the analysis is to quantify the relative abundance of each proteoform within the HomMTM spectrum. Accurate quantification is essential for understanding the biological significance of each variant, as changes in the abundance of specific proteoforms can be indicative of physiological or pathological processes.

Numerous studies have already delved into the computational analysis based on HomMTM spectrum (Wang *et al.* 2010, 2014). For instance, Zhu *et al.* proposed a graph-based algorithm for the identification and quantification of proteoforms. They referred to the maximum *k*-splittable flow (M*k*SF) problem and transformed the HomMTM spectrum quantification problem into a minimum error *k*-splittable flow (ME*k*SF) problem (Zhu and Liu 2018). By constructing a graph that represents all different proteoforms that arise from the same protein, the algorithm can efficiently identify and quantify precise proteoforms with relative abundances in a polynomial time. In these computational analysis algorithms, handling the mass errors of peaks in the input spectrum is a critical issue. Traditional methods use an error tolerance value and approximately regard the masses within this margin of error tolerance value to be equal (Kou *et al.* 2016). However, this could lead to significant error accumulation if positive (or negative) errors occur consecutively (for details, see the Supplementary Material S1). The problem is so severe that some existing software packages include a step to further refine the alignments (Kou *et al.* 2017), indicating the necessity for more efficient error handling strategies.

To address this, Zhan and Wang (2024) proposed a new proteoform identification algorithm for error correction of peaks. They introduced the offset of peaks for imposing the masses to be identical all the time. According to this new algorithm, we can obtain more reliable global alignments within a reasonable time and further overall the quality of proteomics analysis. It is also the first time that global optimal error correction alignments can be obtained using real data sets. Therefore, we think it is not only viable but also highly beneficial to extend this algorithm for the quantification of HomMTM spectra. By adopting the error correction algorithm for HomMTM spectra, we can more accurately account for the unique challenges presented in the multiple proteoforms identification and quantification of HomMTM spectra.

Here, we referred to this error correction algorithm and proposed an algorithm for proteoform quantification based on the top-down tandem mass spectrum. In the combination alignments of the HomMTM spectrum and the reference protein, we need to give a correction of the mass for each matched peak within the pre-defined error range. After the correction, we impose that the mass between *any* two (not necessarily consecutive) matched nodes in the protein is identical to that of the corresponding two matched peaks in the HomMTM spectrum. We design a back-tracking graph to store such kind of information and find a combinatorial path (*k* paths) with the minimum sum of peak intensity error in this back-tracking graph. The obtained alignment can also show the relative abundance of these proteoforms (paths). Our experimental results demonstrate the algorithm’s capability to identify and quantify proteoform combinations encompassing a greater number of peaks. This advancement holds promise for enhancing the accuracy and comprehensiveness of proteoform quantification, addressing a crucial need in the field of top-down MS-based proteomics.

## 2 Materials and methods

**Figure 1.**
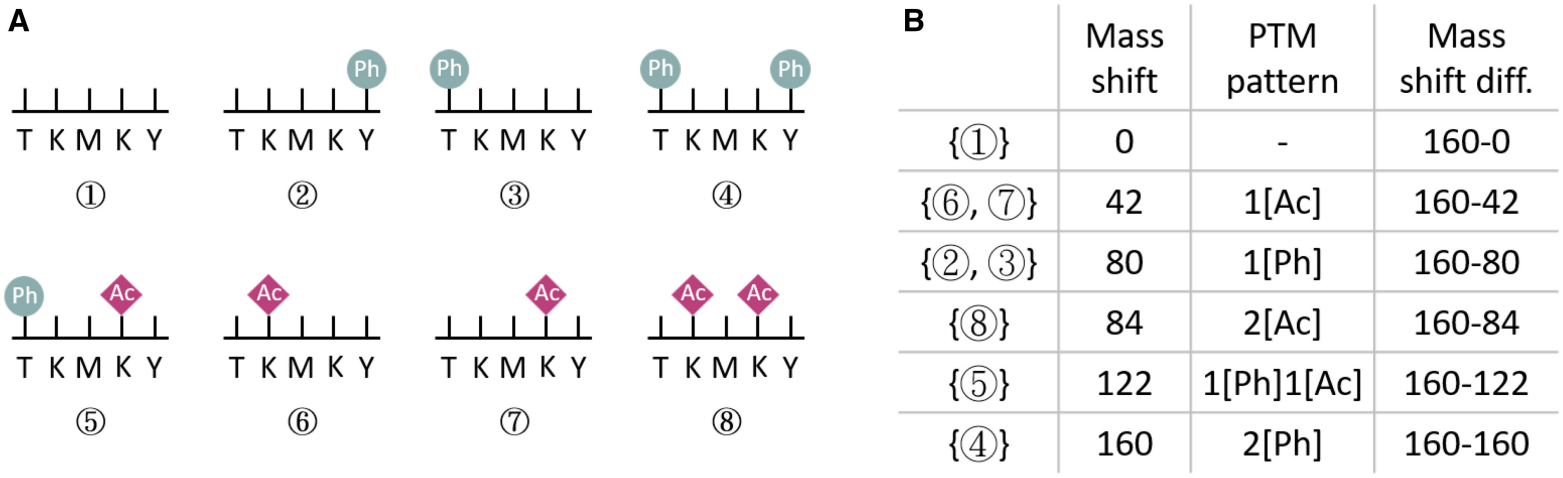
An example of PTM pattern inference problem. (A) Eight possible proteoforms for the protein segment *TKMKY*. (B) The mass shifts, corresponding PTM patterns, and mass shift differences for these eight possible proteoforms.

A protein may have multiple possible proteoforms. For example, phosphorylation (Ph) may occur at the serine (S), threonine (T), or tyrosine (Y) sites and acetylated (Ac) may occur at the lysine (K) sites. An example is given in Fig. 1A, where the original protein segment is *TKMKY*, there are totally 16 possible proteoforms and we list 7 of them as types 2, 3, …, 8 here. The possible mass shifts of those 7 proteoforms are given in Fig. 1B. We can see that a mass shift may correspond to more than one proteoform, e.g. both type 6 and type 7 have one Ac modification (at different locations).

**Figure 2.**
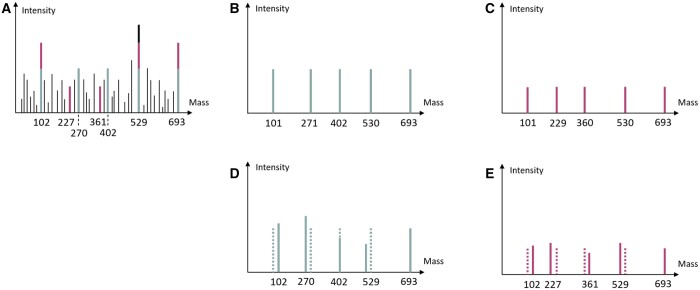
(A) An example HomMTM spectrum containing both types 6 and 7 proteoforms in Fig. 1A. (B) The theoretical spectrum for type 6 proteoform. (C) The theoretical spectrum for type 7 proteoform. (D) The alignment of type 6 proteoform, where solid peaks are real peaks from the input mass spectrum and dotted peaks are theoretical. (E) The alignment of type 7 proteoform, where solid peaks are real peaks from the input mass spectrum and dotted peaks are theoretical. Note that, in both (D) and (E), the real peaks have masses slightly different from that of theoretical peaks. The intensities of real peaks are also slightly different from the theoretical ones.

The mass spectrum generated from the proteoforms of the protein contains a set of peaks with lots of noise, and each peak has a corresponding intensity. Figure 2A is a small example, where black peaks are noises. Each proteoform has a set of theoretical peaks indicating the masses of the segments. The theoretical peaks for types 6 and 7 proteoforms are given in Fig. 2B and C, respectively. To identify a proteoform, we need to find/select peaks from the input mass spectrum that best match the theoretical mass spectrum of those proteoforms. Figure 2D and E are the selected peaks for types 6 and 7 proteoforms. To identify a proteoform, we can obtain a sequence of matched peak pairs, e.g. (101, 102) (271, 270), (402, 402), (530, 529), (693, 693) for type 6 proteoform. Such a sequence of matched pairs is also referred to as an *alignment*. Note that the mass values of peaks from the mass spectrum have errors and the masses of matched peaks are allowed to be slightly different from the theoretical peaks.

### 2.1 Overview of the method

Given a HomMTM spectrum *H*, and its original protein sequence *G*, the task is to find the multiple proteoforms corresponding to the HomMTM spectrum *H* and their corresponding abundance. If the original protein sequence *G* is not known, one can use existing database search methods such as TopMG (Kou *et al.* 2017) or TopMGFast (Zhan and Wang 2024) to obtain *G*.

The first sub-task is to identify all possible proteoforms based on *H* and *G*. Our idea is to extend the dynamic programming algorithms for alignment of a spectrum mass graph with a protein mass graph, where each matched proteoform is represented by an alignment between some peaks in *H* and some nodes in *G* (Kou *et al.* 2017, Zhan and Wang 2024). The dynamic programming algorithms have an efficient way to compute the largest size alignments by using a matrix. The matrix can also be viewed as a graph, where each cell is viewed as a node. In fact, it is well-known that the matrix contains information for all kinds of alignments and thus all kinds of matched proteoforms. Here we design an algorithm to construct a sub-graph named *backtracking graph* based on the matrix, which contains all kind of matched proteoforms interesting to us. Once we have the backtracking graph representing all kinds of possible matched proteoforms, we will try to identify *k* (*k* = 2 in our paper) paths (alignments) in the backtracking graph to get the *k* reliable matched proteoforms by using the peak intensity values, and consequently obtain the values of abundance for the *k* matched proteoforms. The algorithm is given in Section 2.5.

### 2.2 Identification of proteoforms for a given protein

For a given protein sequence, the concept of *proteoform mass graph* (PMG for short) was proposed in Kou *et al.* (2017) to represent all possible proteoforms for the given protein in a concise way. In a PMG of a protein with *n* amino acids, there are *n* + 1 nodes organized from left to right in linear order corresponding to the left and right ends of the *n* amino acids. A black directed edge connecting two neighbor nodes corresponds to an amino acid, with the weight representing the mass of this amino acid. For every potential modification to an amino acid, an additional red edge is added with the modified mass between corresponding nodes. A PMG stores the corresponding normal protein with all its possible proteoforms.

A spectrum is formulated as a *spectrum mass graph* (SMG for short). In an SMG, each node corresponds to a unique peak, with the associated mass assigned as the weight. A directed edge is added between a pair of nodes, with its weight as the difference in the weights of the two corresponding nodes.

To simplify the further analysis, we transformed all edges in PMG and MMG into integers through scaling and rounding techniques (Liu *et al.* 2013).

Traditionally, the problem of finding a proteoform of a given protein is formulated as finding *an alignment* between the two graphs.

#### 2.2.1 Alignment of PMG and SMG

Let *x*_0_, *x*_1_, …, *x_n_* be the *n* nodes in a PMG *G* and *y*_0_, *y*_1_, …, *y_m_* be the *m* nodes in a MMG *H*. We use *m_i_* to represent the mass value of node/peak *y_i_* in *H*. *M* = *m_m_* is the mass value of the proteoform corresponding to the input mass spectrum. Let *δ* be a user defined number for error tolerance. An alignment of *G* and *H* with length *s* is a sequence of matched pairs $\left(x_{j_{1}},y_{i_{1}}),\;(x_{j_{2}},y_{i_{2}}),\;\ldots ,\;(x_{i_{s}},y_{j_{s}}\right)$, where $0\;\leq \;j_{1}<j_{2}<\ldots ,j_{s}\;\leq \;n,0\;\leq \;i_{1}<i_{2}<\ldots ,i_{s}\;\leq \;m$, and there exists a path between node $x_{i_{t}}$ and node $x_{i_{t-1}}$ in *G* with total mass in the range $\left[m_{i_{t}}-m_{i_{t-1}}-\delta ,m_{i_{t}}-m_{i_{t-1}}+\delta \right]$ for t=2,3,…,s.

In practice, we impose the condition that $y_{i_{1}}=y_{0}$ and $y_{i_{s}}-y_{m}$ to match the whole mass spectrum to a segment in *G*.

The above alignment definition imposes that the mass difference between two consecutive matched peaks is roughly the same (with error tolerance value *δ*) as the mass of the corresponding sub-path in *G*. This method may suffer from error accumulation, where *k* consecutive positive/negative errors in the alignment will lead to an error of kδ between a pair of peaks in *H*. This is certainly evidence that such an alignment is not reliable.

To get more realistic alignments, Zhan and Wang (2024) proposed a model considering peak error correction.

#### 2.2.2 Peak error correction alignment

A length *r* peak error correction alignment for a PGM *G* and SMG *H* is a sequence of triples $\left(x_{j_{1}},y_{i_{1}},k_{i_{1}}),(x_{j_{2}},y_{i_{2}},k_{i_{2}}),\;\cdots ,\;(x_{j_{r}},y_{i_{r}},k_{i_{r}}\right)$, where $k_{i_{q}}\in (-\delta ,\delta )$ is the error correction value for peak $y_{i_{q}}$ such that $m_{i_{q}}+k_{i_{q}}-(m_{i_{q-1}}+k_{i_{q-1}})=M_{j_{q-1},j_{q}}$, where $m_{i_{q}}$ is the mass for the peak $y_{i_{q}}$ and $M_{j_{q-1},j_{q}}$ is the mass of a path between node $x_{j_{q-1}}$ and node $x_{j_{q}}$. Note that *δ* represents the error tolerance value for the mass of a peak. The *proteoform identification problem* is to find a set of error correction alignments with the maximum alignment size between *G* and *H*.

We denote the practical peak intensity of the node/peak *y_i_* in *H* as *p*(*i*). The *most similar alignment* for an alignment obtained from the individual alignment problem is an alignment with the theoretical relative abundance *h* such that the sum of peak intensity error between *h* and *p*(*i*) is minimized.

### 2.3 An algorithm for identification of proteoforms

To obtain a set of proteoforms best fitting the SMG, we want to compute the longest length peak error correction alignment between a PMG and an SMG. The following algorithm was proposed by Zhan and Wang (2024).

Let T(i,j,k) be the maximum size of an error correction alignment between the first *i* peaks in *H* and the first *j* nodes in *G* such that the corrected peak of *y_i_* has the mass value $m_{i}+k$ and *y_i_* in *H* matches *x_j_* in *G*. When computing T(i,j,k) for every 0 ≤ i ≤ m, 0 ≤ j ≤ n and $-\delta_{i}\;\leq \;k\;\leq \;\delta_{i}$, a dynamic programming algorithm can be used to simplify the process. Here they re-compute the *error correction range* of *k_i_* to be $\left[-\delta_{i}^{-},\delta_{i}^{+}\right]$ for every peak *y_i_* to eliminate redundant calculations when computing T(i,j,k), since there may exist an overlap between the error correction ranges of some close peaks.

Let *d*(*s*, *j*) denote the set of distinct path masses from node *x_s_* to node *x_j_* in *G*. For each mass m∈d(s,j), there exists a list of nodes in *H* that corresponds to peaks with mass values within the range $\left[(m_{i}+k)-m-\delta_{\max},(m_{i}+k)-m+\delta_{\max}\right]$. Define list(i,j,k,m) as the sorted list of such nodes. These sorted nodes can be aligned with node *x_s_* in *G*.

The following equation is used for computing T(i,j,k).

(1) $$\begin{array}{c} T(i,j,k)=\overset{j\prime =0}{\underset{j\prime =j-1}{\max}}\;\;\;\underset{m\in d(j\prime ,j)}{\max}\\ \underset{\left(i\prime ,k\prime )\;\mathrm{satisfying}\;(2\right)}{\max}\{T(i\prime ,j\prime ,k\prime )+1\}, \end{array}$$

where condition (2) is as follows:

(2) $$\;m-[(m_{i}+k)-m_{i\prime }]=k\prime .$$

### 2.4 Constructing backtracking graph

In order to find all possible proteoforms that best fit the input mass spectrum, we constructed a graph during the backtracking process. Note that, we are interested in alignments ending with *y_m_*, the last peak with mass value *M* = *m_m_*. Thus, we will find a set E containing all cells T(m,j,k) with the largest value among all cells T(m,j,k) for j=1,2,…,n. If there exist *k* and k′ such that T(m,j,k)=T(m,j,k′), We only need to consider one of *k* and k′. The reason is that the range of *k* and k′ is much smaller than the mass value of a possible (modified or unmodified) amino acid and different *k* values cannot lead to different optimal alignments (see Zhan and Wang 2022). We can do backtracking starting with all the cells T(m,j,k) in E. For each preceding cell T(i′,j′,k′) used in Equation (1) that leads to the maximum value of T(i,j,k), we treat both T(i′,j′,k′) and T(i,j,k) as nodes in the backtracking graph and add a directed edge from T(i′,j′,k′) to T(i,j,k). Note that for a fixed T(i,j,k), there may exist more than one T(i′,j′,k′) leading to the maximum value of T(i,j,k). The backtracking graph contains all those T(i′,j′,k′)s. The backtracking process stops when no preceding cell can be found. We then prune the backtracking graph *B* so that only nodes that are the descendants of cells T(m,j,k) will be kept in the backtracking graph. The above method will construct a backtracking graph *B* containing all proteoforms with mass *M* = *m_m_*. Since each node in *B* has a T() value, we use the T() values to decompose the nodes in *B* into layers, where layer *t* contains all the nodes with T()=t.

#### 2.4.1 Two proteoforms have different total mass

In practice, the input mass spectrum may also contain peaks from different proteoforms of the same protein with total mass different from *M*. In that case, the total mass values of the different proteoforms should be *M − x*, where *x* (positive number) is the difference between two mass shifts. For example, in Fig. 1B, if*M* is the mass for the type 4 proteoform, then x=160−122 and the mass value for type 5 proteoform is M−(160−122). Given a protein sequence and its possible modifications, we can easily construct the list of mass shift differences. See Fig. 1B (the rightmost column) as an example.

In this case, we will start with a node T(m,j,k) in E and the other node T(i′,j′,k′) such that $m_{i\prime }$ is in the range [M−x−δ,M−x+δ] and T(i′,j′,k′) is the largest to construct the backtracking graph. Similarly, we organize all the nodes in the backtracking graph layer by layer based on their T() values.

A small example is given in the Supplementary Material S1.

### 2.5 Proteoform quantification

We compute peak error correction alignments that best fit the input mass spectrum to identify proteoforms. When a mass spectrum contains multiple proteoforms, we want to find the abundances of each proteoform. The intensities of matched peaks in the corresponding alignments can be used for proteoform quantification. Here, we consider the case, where there are two proteoforms.

Let $A=(x_{j_{1}},y_{i_{1}},k_{i_{1}}),\;(x_{j_{2}},y_{i_{2}},k_{i_{2}}),\;\cdots ,\;(x_{j_{r}},y_{i_{r}},k_{i_{r}})$ be the error correction alignment for a proteoform *P*. Let *p* be the theoretical peak intensity for the proteoform. The total intensity error for the proteoform is defined as $e(A)=\sum_{t=1}^{r}|p-p_{i_{t}}|$, where $p_{i_{t}}$ is the intensity of peak $y_{i_{t}}$.

Intuitively, the proteoform quantification problem is to find two alignments *A*1 and *A*2 from the backtracking graph and the corresponding theoretical intensities *p*_1_ and *p*_2_ such that $e(A_{1})+e(A_{2})$ is minimized. The abundance of the proteoform corresponding to *A_i_* is $\frac{p_{i}}{p_{1}+p_{2}}$ for *i* = 1 and 2.

Since the two proteoforms may share some common peaks, we have to define the intensity error for a pair of nodes in the backtracking graph. We consider two cases.

Case 1: The two proteoforms have the same total mass *M*. Let $v_{a}=T(i_{a},j_{a},k)$ and $v_{b}=T(i_{b},j_{b},k\prime )$ be two nodes in *B* at the same layer.

(3) $$\varepsilon (v_{a},v_{b})=\{\begin{array}{cc} \left|p(v_{a})-p_{1}-p_{2}\right| & \text{if}\;v_{a}=v_{b};\\ \left|p(v_{a})-p_{1}|+|p(v_{b})-p_{2}\right| & \text{otherwise}. \end{array}$$

where $p(v_{a})$ and $p(v_{b})$ represent intensity of the peaks $y_{j_{a}}$ and $y_{j_{b}}$, respectively.

Let $D(v_{a},v_{b})$ be the minimum sum of intensity errors for two paths from a node T(m,j,k) in E (last layer) to *v_a_* and *v_b_*, respectively, where *v_a_* and *v_b_* are at the same layer before T(m,j,k). We can compute $D(v_{a},v_{b})$ as follows:

(4) $$D(v_{a},v_{b})=\varepsilon (v_{a},v_{b})+\underset{v\prime_{a}\in f(v_{a}),v\prime_{b})\in f(v_{b})}{\min}D(v\prime_{a},v\prime_{b}).$$

where *f*(*v*) is the set of parent nodes of *v*. Note that $v\prime_{A}$ and $v\prime_{b}$ are at the same layer by definition.

Note that *p*_1_ and *p*_2_ are not known. Let *p_max_* be the maximum peak intensity for all the peaks involved in *B*. We can guess the values of both *p*1 and *p*_2_ as $p_{\max}\times x\%$, where x=1,2,…,100.

The algorithm is described as Algorithm 1.

Algorithm 1.
The algorithm to the combinatorial alignment problem.

1: **for**$p_{1}=p_{\max}\times 1\%\to p_{\max}\times 99\%$**do**

2:   **for**$p_{2}=p_{\max}\times 1\%\to p_{\max}\times 99\%$**do**

3:    compute $D(v_{a},v_{b})$ layer by layer.

4:   **end for**

5: **end for**

Case 2: The two proteoforms have total masses *M* and *M − x* for *x* > 0, respectively. In this case, *x* should be a given mass shift difference based on the input protein and its possible modifications. An example is given in Fig. 1B.

In order to obtain two proteoforms under Case 2, we will try to find two alignments corresponding to two paths from the two starting backtracking nodes T(m,j,k) and T(i′,j′,k′) back to a node T(0,j″,k″). The two paths may have different numbers of edges due to various reasons, e.g. loss of peaks, etc.

Without loss of generality, we assume that T(m,j,k)>T(i′,j′,k′). (The other case is similar.) For any pair of nodes *v_a_* and *v_b_* in the same layer i, for i=1,2,…,T(i′,j′,k′), we still use Equations (3) and (4) to compute $\varepsilon (v_{a},v_{b})$ and $D(v_{a},v_{b})$.

Node that the starting node $T(i\prime ,j\prime ,k\prime )=v_{b}$ is at layer T(i′,j′,k′). For any pair of nodes *v_a_* and *v_b_* at layer T(i′,j′,k′), we need to modify the way to calculate $\varepsilon (v_{a},v_{b})$.

$$\varepsilon (v_{a},v_{b})=\{\begin{array}{cc} \left|p(v_{a})-p_{1}-p_{2}|+d(v_{b}\right) & \text{if}\;v_{a}=v_{b};\\ \left|p(v_{a})-p_{1}|+|p(v_{b})-p_{2}|+d(v_{a}\right) & \text{otherwise}, \end{array}$$

where *d*(*v*) is the minimum total peak error among all the paths from T(m,j,k) to the parents of *v* in the backtracking graph and *d*(*v*) can be calculated as follows:

(5) $$d(v)=\underset{v\prime \in f(v)}{\min}|p(v\prime )-p_{1}|+d(v\prime )$$

(6) d(v)=0 if v is T(m,j,k).

## 3 Results

First, we use simulated datasets to evaluate the prediction quality.

### 3.1 Prediction quality evaluation

To evaluate the quality of the proteoform quantification algorithm, we generate the simulated top-down HomMTM spectra using Top-down MaSS-Simulator, a package specifically designed to generate top-down spectra, based on selected protein sequences (see https://github.com/KunyiE1/TopDown-MaSS-Simulator).

To get the selected protein sequences, we randomly select 10 proteins from the whole Histone H4 protein database used in Liu *et al.* (2013) downloaded from UniProt (Accession number: P62805) including 291 protein entries. Then we use these 10 proteins to generate the simulated top-down HomMTM spectra and the size of amino acids contained in each protein is from 65 to 109.

Three mutations were used as variable PTMs: lysine (K) to cysteine (C) (UNIMOD Accession number: 1132), threonine (T) to alanine (A) (UNIMOD Accession number: 659), and valine (V) to glycine (G) (UNIMOD Accession number: 672). These three mutations were also gathered together in a txt format file as input. Note that the total masses of these two different proteoforms may be the same or differ by some mass shifts according to Case 1 and Case 2 described in Methods. In this way, for each of the simulated top-down HomMTM spectra, we know the real corresponding protein as well as the corresponding proteoforms.

#### 3.1.1 Generating mixture top-down HomMTM spectra

To generate the top-down HomMTM spectra using the website https://github.com/KunyiE1/TopDown-MaSS-Simulator, we randomly select one kind of proteoform for the selected protein and convert it to the corresponding sequence file. Then we run the simulation program and get a simulated top-down spectrum file for the proteoform of the protein. To add peaks of the other proteoform of the same protein, we manually calculate the mass values of the theoretical peaks for the other kind of proteoform for the same protein, add some random mass errors within the error tolerance setting, and add these peaks to the simulated top-down spectrum file. Besides, we set the mean value of the peak intensities of one kind of proteoform to be *x* = 500 and the other kind of proteoform to be *x*, 2*x*, and 3*x*, respectively. The variances of the peak intensities are set to be 50 and 25, respectively. Therefore, for this simulated mixture top-down HomMTM spectra, the theoretical relative abundance of these two proteoforms should be 50% vs 50%, 33.3% vs 66.7%, and 25% vs 75%, respectively.

#### 3.1.2 Identification and quantification results

For each mixture top-down HomMTM spectrum, we apply our method to directly align the spectrum with the corresponding protein. The detailed results are shown in Table 1. We can see that our algorithm can report the two correct proteoforms for all the simulated mixture HomMTM top-down spectra and the relative abundances for most proteoforms are also correct.

**Table 1.** Proteoform identification and quantification results for simulated top-down HomMTM spectrum.

Protein	Size	# of peak1^a^	# of peak2^a^	# of mod1^b^	# of mod2^b^	Abundance	Running time (ms)
sp|P0AB83|END3_ECOLI	65	27	23	0	3	59% and 41%	2316.09
sp|P0ABU0|MENB_ECOLI	68	18	17	0	3	50% and 50%	1166.22
sp|P77690|ARNB_ECOLI	74	16	14	0	3	51% and 49%	1007.79
sp|P0A948|RIMJ_ECOLI	77	14	23	0	3	41% and 59%	957.40
sp|P37626|YHII_ECOLI	85	23	18	0	3	67% and 33%	1462.98
sp|P0DP22|YJIQ_ECOLI	88	9	15	0	3	56% and 43%	785.56
sp|P0AF03|MOG_ECOLI	91	18	24	0	3	58% and 42%	1752.53
sp|P10441|LPXB_ECOLI	98	19	20	0	3	70% and 30%	1725.71
sp|P05458|PTRA_ECOLI	103	13	13	0	3	12% and 88%	1004.92
sp|P0AFS1|LSRD_ECOLI	109	17	20	0	3	78% and 22%	1589.71

a These two columns indicate the total numbers of matched peaks when aligning the mixture top-down HomMTM spectrum with the first and second proteoforms, respectively.

b These two columns indicate the total numbers of modifications in the first and second proteoforms, respectively.

#### 3.1.3 Evaluation on mixture bottom-up HomMTM spectra

We also evaluate the quality of the proteoform quantification algorithm on the simulated bottom-up HomMTM spectra. For more experiment details and results, see the Supplementary Material S1.

### 3.2 Case study

**Figure 3.**
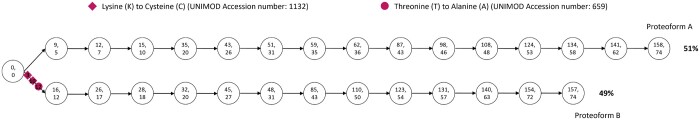
The graphical illustration of the quantification results for case 2. The original protein is sp|P77690|ARNB_ECOLI with 74 amino acids. The two numbers on each nodes represent the peak number and node number in the alignment, respectively.

Here give a detailed explanation for the case in the third row of Table 1, which is the protein sp|P77690|ARNB_ECOLI with 74 amino acids. Figure 3 shows the detailed graphical illustration of the quantification results.

As shown in Fig. 3, two proteoforms are identified and they are represented as two paths. For the proteform *A*, there is no reported PTM. For the proteoform *B*, the first PTM lysine (K) to cysteine (C) reported to occur in the third amino acid, the second PTM lysine (K) to cysteine (C) reported to occur in the 10th amino acid and the last PTM hreonine(T) to alanine (A) reported to occur in the 12th amino acid. Since these two proteoforms have different total masses, the ending node pairs of these two proteoforms are different. In addition, the abundance of the proteoform *A* is 51% and the abundance of the proteoform *B* is 49%. Under the condition of this combinatorial abundance, these two proteoforms have the minimum sum of peak intensity errors. Besides, this spectrum can align 15 more peaks when identified as these two combinatorial proteoforms than when identified as a single proteoform.

### 3.3 Results on real datasets

In this section, we apply our method to some real datasets. The mass spectra dataset is generated from the human Histone H4 protein downloaded from Liu *et al.* (2013). The reference protein database is download from UniProt (Liu *et al.* 2013) and it includes 291 reference proteins. For this Histone H4 dataset (database), two mutations were used as variable PTMs similar to Liu *et al.* (2013) and these two modifications are Phosphorylation (UNIMOD Accession number: 21) and di-Methylation (UNIMOD Accession number: 36). A txt format file was generated based on these two pre-defined mutations as part of the input.

#### 3.3.1 Comparison with Zhu and Liu’s algorithm

Here, we compared our method with Zhu and Liu’s method proposed in Zhu and Liu (2018) using the Histone H4 real datasets introduced above. As suggested in Zhu and Liu (2018), an alignment with at least 10 matched peaks is considered reliable. With a cutoff of 10 matched peaks, our algorithm identified 5 spectra matched to single (reliable) proteoform and 1154 spectra matched to (reliable) proteoform pairs while Zhu and Liu’s algorithm identified 441 spectra matched to single proteoforms and 184 spectra matched to proteoform pairs.

To show some details, we select 10 mass spectra and used our method to find proteoforms. For each of the 10 cases, we have found two proteoforms and their corresponding relative abundances. The details are given in the Table 2.

**Table 2.** Proteoform identification and quantification results for real top-down HomMTM spectrum.

Protein	Size	# of peak1^a^	# of peak2^a^	# of mod1^b^	# of mod2^b^	Abundance	Running time (ms)
sp|P0DPK2|H3Y1_HUMAN	94	16	16	23	23	50% and 50%	85 692.89
sp|O00213|APBB1_HUMAN	101	13	13	10	10	41% and 59%	44 631.51
sp|O75461|E2F6_HUMAN	93	18	18	18	18	54% and 46%	52 947.28
sp|O00268|TAF4_HUMAN	37	9	9	7	9	38% and 62%	23 077.81
sp|P0DPK5|H3Y2_HUMAN	95	17	17	19	18	47% and 53%	37 206.15
sp|Q03164|KMT2A_HUMAN	93	27	27	16	15	50% and 50%	2 61 174.37
sp|Q9P0M6|H2AW_HUMAN	102	21	21	25	27	54% and 46%	2 41 834.38
sp|Q14839|CHD4_HUMAN	91	19	19	27	28	42% and 58%	80 985.51
sp|Q93077|H2A1C_HUMAN	101	17	17	8	9	36% and 64%	79 641.00
sp|Q4FZB7|KMT5B_HUMAN	96	21	21	22	22	52% and 48%	48 161.31

a These two columns indicate the total numbers of matched peaks when aligning the mixture top-down HomMTM spectrum with the first and second proteoforms, respectively.

b These two columns indicate the total numbers of modifications in the first and second proteoforms, respectively.

## 4 Conclusion

We proposed an algorithm for proteoform quantification with peak error corrections based on top-down tandem mass spectrum and produced a program package in C++. Our experimental results on real-world data demonstrate the capability to quantify proteoform combinations with at most two different proteoforms. This is a small step for proteoform quantification, which is very much needed in the field of top-down MS-based proteomics. Still, it is an interesting open problem to design algorithms for identifying and quantifying mixture spectra with larger number of proteoforms.

## Supplementary Material

btaf007_Supplementary_Data

## Data Availability

All source code and experiment data are available at https://github.com/Zeirdo/TopMGQuant.

## References

[btaf007-B1] Cesnik AJ , Shortreed MR, Schaffer LV et al Proteoform suite: software for constructing, quantifying, and visualizing proteoform families. J Proteome Res 2018;17:568–78. 29195273 10.1021/acs.jproteome.7b00685 PMC5770237

[btaf007-B2] DiMaggio PA , Young NL, Baliban RC et al A mixed integer linear optimization framework for the identification and quantification of targeted post-translational modifications of highly modified proteins using multiplexed electron transfer dissociation tandem mass spectrometry. Mol Cell Proteomics 2009;8:2527–43. 19666874 10.1074/mcp.M900144-MCP200 PMC2773719

[btaf007-B3] Kafader JO , Melani RD, Durbin KR et al Multiplexed mass spectrometry of individual ions improves measurement of proteoforms and their complexes. Nat Methods 2020;17:391–4. 32123391 10.1038/s41592-020-0764-5 PMC7131870

[btaf007-B4] Kou Q , Wu S, Tolić N et al A mass graph-based approach for the identification of modified proteoforms using top-down tandem mass spectra. Bioinformatics 2017;33:1309–16. 28453668 10.1093/bioinformatics/btw806 PMC5860502

[btaf007-B5] Kou Q , Xun L, Liu X. Toppic: a software tool for top-down mass spectrometry-based proteoform identification and characterization. Bioinformatics 2016;32:3495–7. 27423895 10.1093/bioinformatics/btw398 PMC5181555

[btaf007-B6] Liu X , Hengel S, Wu S et al Identification of ultramodified proteins using top-down tandem mass spectra. J Proteome Res 2013;12:5830–8. 24188097 10.1021/pr400849y PMC3905687

[btaf007-B7] Schaffer LV , Millikin RJ, Miller RM et al Identification and quantification of proteoforms by mass spectrometry. Proteomics 2019;19:e1800361. 31050378 10.1002/pmic.201800361 PMC6602557

[btaf007-B8] Wang J , Bourne PE, Bandeira N. Mixgf: spectral probabilities for mixture spectra from more than one peptide. Mol Cell Proteomics 2014;13:3688–97. 25225354 10.1074/mcp.O113.037218 PMC4256515

[btaf007-B9] Wang J , Pérez-Santiago J, Katz JE et al Peptide identification from mixture tandem mass spectra. Mol Cell Proteomics 2010;9:1476–85. 20348588 10.1074/mcp.M000136-MCP201 PMC2938093

[btaf007-B10] Zhan Z , Wang L. Proteoform identification based on top-down tandem mass spectra with peak error corrections. Brief Bioinform 2022;23:bbab599. 35136947 10.1093/bib/bbab599

[btaf007-B11] Zhan Z , Wang L. Fast peak error correction algorithms for proteoform identification using top-down tandem mass spectra. Bioinformatics 2024;40:btae149. 38498847 10.1093/bioinformatics/btae149 PMC11212493

[btaf007-B12] Zhu K , Liu X. A graph-based approach for proteoform identification and quantification using top-down homogeneous multiplexed tandem mass spectra. BMC Bioinformatics 2018;19:280–4. 30367573 10.1186/s12859-018-2273-4 PMC6101081

