# Supplementary material for: Proteoform identification and quantification based on alignment graphs
Source: Bioinformatics. 2025 Jan 9;41(1):btaf007. doi: 10.1093/bioinformatics/btaf007 (PMC11769674; doi:10.1093/bioinformatics/btaf007)
Supplement: btaf007_Supplementary_Data [file btaf007_supplementary_data.zip › 179fe_supplementary material.pdf]

Supplementary Material

1. PEAK ERROR ACCUMULATION

Let (x_j, x_i) and $(x_{j'}, y_{i'})$ be the two consecutive matched pairs in the alignment. They allow the mass between x_j and $x_{j'}$ and the mass between peaks y_i and $y_{i'}$ to differ by at most δ_i. However, the error accumulation of this method may lead to inaccurate alignment.

Here is an example. Figure S1 illustrates a PMG with nodes $x_0, x_1, \cdots, x_5$, where there is a modification on each of the three edges (x_1, x_2), (x_2, x_3) and (x_3, x_4), and a SMG with nodes $y_0, y_1, \cdots, y_5$. The exact masses of the peaks are 0, 76, 279, 426, 612, 732, respectively. When aligning these two mass graphs, we set the error tolerance δ to be 5 for all the peaks. Therefore, node y_0 can be aligned to node x_0 and node y_1 can be aligned to node x_1 since the difference between the masses of the paths $x_0 \to x_1$ and $y_0 \to y_1$ is $76 - 71$ which is less than or equal to δ. Similarly, by checking the masses at two consecutive pairs, every y_i can be aligned with x_i for $i = 0, 1, \ldots, 5$. However, the mass between x_0 and x_5 (containing the 3 red edges) is 708, while the mass between y_0 and y_5 is 723. The difference between 723 and 708 is 15 which is much larger than $\delta = 5$. Apparently, such an alignment is not reliable.

In fact, this problem is serious and the software package in [1] includes a step to refine the alignments obtained from the dynamic programming algorithm. Therefore, we use the error correction alignment to have more accurate alignments.

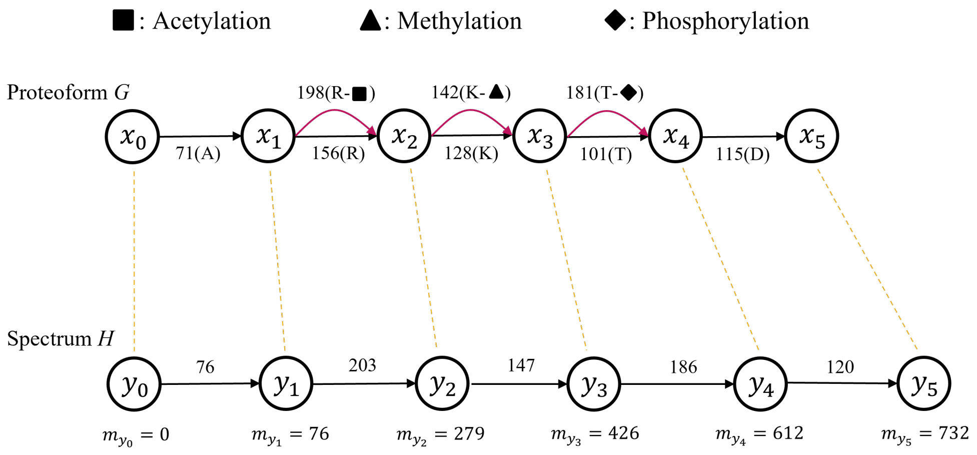

Fig. S1. An alignment example. If an alignment has the alignment errors all positive or all negative like this example, the final output will be different and not convincing.

2. BACKTRACKING GRAPH

To illustrate the backtracking graph construction, we use a small example. Let H be a spectrum containing p_0, p_1, ..., p_7 peaks with the corresponding masses $m_0 = 0$, $m_1 = 101$, $m_2 = 229$, $m_3 = 271$, $m_4 = 360$, $m_5 = 402$, $m_6 = 530$, and $m_7 = 695$. The eight peaks form the two proteoforms given in Fig. 2 (b) and (c), respectively. For simplicity, we assume that the peaks have no error, i.e., $\delta = 0$. The protein mass graph G for protein sequence $TKMKY$ contains six nodes. The matrix $T(i, j)$ with $\delta = 0$ obtained from the dynamic programming algorithm is given in Fig. S2. The last cell $T(7, 5)$ has a value 6 indicating that all the alignments ending at $T(7, 5)$ have 6 matched pairs. For the backtracking process, we start with $T(7, 5)$ and try to go back to all the possible previous cells (nodes) that lead to the optimum value of $T(7, 5)$. In this case, there is only one previous cell $T(6, 4)$. For node $T(6, 4)$, there are two possible previous cells, $T(5, 4)$ and $T(4, 4)$, that lead to the optimal value 5 of cell $T(6, 4)$. The backtracking graph is indicated by the set of blue nodes and the set of blue edges.

Note that, the backtracking graph contains all possible alignments corresponding to the candidate proteoforms for the input spectrum. In general, the number of possible proteoforms in the backtracking graph is still very large and we need to have a way to find the two (we assume that there are at most two proteoforms for each input spectrum) real proteoforms and their corresponding abundance. The algorith for this step is given in subsection "Proteoform Quantification", where we basically want to find two paths in the backtracking graph such that the intensity error of corresponding peaks is minimized.

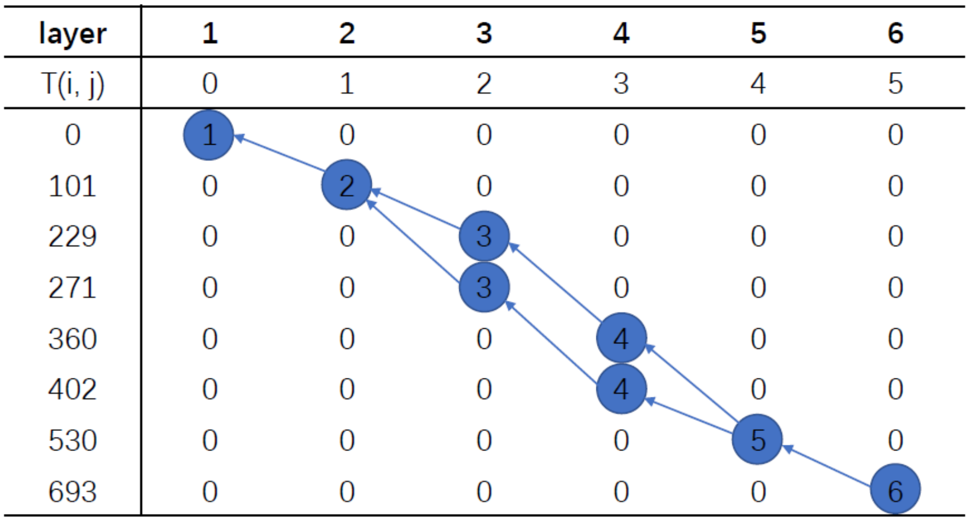

Fig. S2. An example of a backtracking graph.

3. RUNNING TIME OF THE ALGORITHMS

To compute $T(i, j, k)$ and construct the backtracking graphs, the running time is the same as that in [2]. Let n be the total number of nodes in the backtracking graph, q the maximum number of nodes in a layer in the backtracking graph. We need to compute $O(nq)$ $D(v_a, v_b)$'s and each takes $O(q^2)$ time. Thus, the total time for computing $D(v_a, v_b)$ is $O(nq^3)$. Since the input of our method contains the spectrum and a protein mass graph only (instead of the whole database), it is very fast in practice comparing to the time required to database search.

4. PREDICTION QUALITY EVALUATION ON BOTTOM-UP HOMMTM SPECTRUM

To evaluate the quality of the proteoform quantification algorithm, we generate the simulated bottom-up HomMTM spectra using MaSS-Simulator [3] based on selected peptide sequences.

To get the selected peptide sequences, we randomly select 20 proteins from the whole Histone H4 protein database used in[4] and downloaded from UniProt (Accession number: P62805) including 291 protein entries. We randomly select a segment from each selected protein to generate the simulated bottom-up HomMTM spectra.

According to [5], the size of the bottom-up spectrum should be from 7 to 21 amino acids in general. The 20 selected peptide sequences contain 7 to 21 amino acids. Two mutations were used as variable PTMs and those two modifications are Phosphorylation (UNIMOD Accession number: 21) and Di-Methylation (UNIMOD Accession number: 36). Note that the total masses of these two different proteoforms may be the same or differ by some mass shifts according to Case 1 and Case 2 described in Methods. In this way, for each of the simulated bottom-up HomMTM spectra, we know the real corresponding peptide as well as the corresponding proteoforms.

Generating mixture bottom-up HomMTM spectra: To generate the bottom-up HomMTM spectra using the MaSS-Simulator, we randomly select one kind of proteoform for the selected peptide and convert it to the corresponding sequence file. Then we run the software MaSS-Simulator and get a simulated bottom-up spectrum file for the proteoform of the peptide. To add peaks of the other proteoform of the same peptide, we manually calculate the mass values of the theoretical

peaks for the other kind of proteoform for the same peptide, add some random mass errors within the error tolerance setting, and add these peaks to the simulated bottom-up spectrum file. Besides, we set the mean value of the peak intensities of one kind of proteoform to be $x = 1000$ and the other kind of proteoform to be x, $0.5x$ and $0.25x$, respectively. The variances of the peak intensities are set to be 50 and 25, respectively. Therefore, for this simulated mixture bottom-up HomMTM spectra, the theoretical relative abundance of these two proteoforms should be 50% vs 50%, 66.7% vs 33.3%, and 80% vs 20%, respectively.

Identification and quantification results: For each mixture bottom-up HomMTM spectrum, we apply our method to directly align the spectrum with the corresponding peptide. The detailed results are shown in Table S1. We can see that our algorithm can report the two correct proteoforms for all the simulated mixture HomMTM bottom-up spectra and the relative abundances for most proteoforms are also correct.

A. Case Study

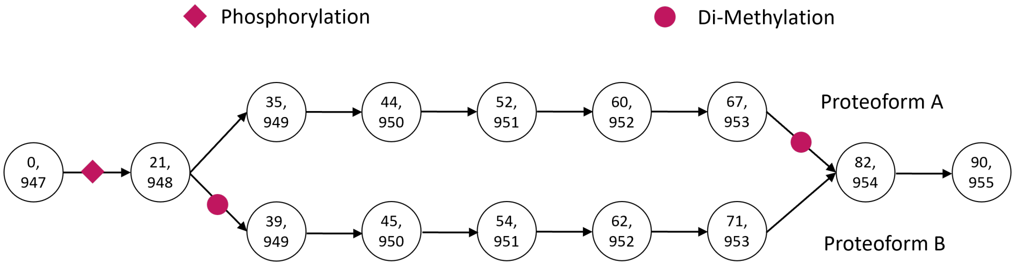

Fig. S3. The graphical illustration of the quantification results for case 1. Proteoforms are Y^1R^2SSDGRT and Y^1RSSDGRT. The two numbers on each nodes represent the peak number and node number in the alignment, respectively.

Here, we choose two small cases to illustrate the details.

For case 1, a peptide with 8 amino acids is randomly selected from the protein $sp \mid Q7Z3B3 \mid KANL1\_HUMAN$ and two proteoforms (Y^1R^2SSDGRT and Y^1RSSDGRT) are constructed using the modifications Phosphorylation (UNIMOD Accession number: 21) and Di-Methylation (UNIMOD Accession number: 36). Fig. S3 shows the detailed graphical illustration of the quantification results using the algorithm we proposed for this small size case.

As shown in Fig. S3, two proteoforms are represented as two paths. A number pair written in a node represents a matched pair of a proteoform graph and a spectrum graph. The first and second numbers represent the peak ID and the residue ID, respectively. Besides, two mutations, Phosphorylation (UNIMOD Accession number: 21) and Di-Methylation (UNIMOD Accession number: 36), are used as variable PTMs. The result PTMs are also illustrated on the corresponding edges. For example, for the sub-alignment from the node pair $(0, 947)$ to the node pair $(21, 948)$ in both proteoforms, there exists a shared PTM Phosphorylation. The second PTM occurs in different residues for these two proteoforms, one is from the node pair $(67, 953)$ to the node pair $(82, 954)$ and the other is from the node pair $(21, 948)$ to the node pair $(39, 949)$. Since these two proteoforms have the same total masses, they share the identical ending node pair $(90, 955)$.

In addition, we can also obtain the corresponding abundances for these two proteoforms. The

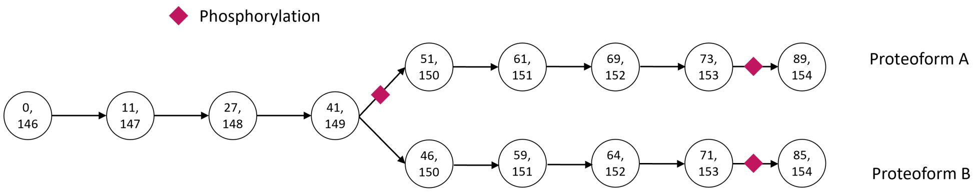

Fig. S4. The graphical illustration of the quantification results for case 2. Proteoforms are $VLQT^1NDLT^1$ and $VLQTNDLT^1$. The two numbers on each nodes represent the peak number and node number in the alignment, respectively.

Table S1. Proteoform identification and quantification results for simulated bottom-up HomMTM spectrum.

Protein	Size	Proteoform	Case	Ab1	RT1 (ms)	Ab2	RT2 (ms)	Ab3	RT3 (ms)
sp|Q8IW19|APLF_HUMAN	7	EIAK^{2}T^{1}QM<br>EIAK^{2}TQM	2	48%<br>52%	1906.3	64%<br>36%	2035	81%<br>19%	1565.1
sp|Q96EZ8|MCRS1_HUMAN	8	VLQT^{1}NDLT^1<br>VLQTNDLT^1	2	50%<br>50%	254.7	65%<br>35%	167.5	80%<br>20%	211.5
sp|Q7Z3B3|KANL1_HUMAN	8	Y^{1}R^{2}SSDGRT<br>Y^{1}RSSDGR^{2}T	1	50%<br>50%	389.2	68%<br>32%	342.2	80%<br>20%	393.5
sp|Q7Z3B3|KANL1_HUMAN	9	MNT^{1}S^{1}GQTAL<br>MNTS^{1}GQT^{1}AL	1	50%<br>50%	456.1	68%<br>32%	367.6	78%<br>22%	354
sp|Q8WUI4|HDAC7_HUMAN	10	WAAGS^{1}VT^{1}DLA<br>WAAGS^{1}VTDLA	2	48%<br>52%	243.7	68%<br>32%	338.8	80%<br>20%	289.1
sp|Q96RI1|NR1H4_HUMAN	11	LT^{1}CEGCK^{2}GFFR<br>LT^{1}CEGCKGFFR^2	1	50%<br>50%	343.1	66%<br>34%	200.4	80%<br>20%	390.9
sp|Q9BZK7|TBL1R_HUMAN	12	HCIR^{2}EGGQDVPS^1<br>HCIR^{2}EGGQDVPS	2	60%<br>40%	1664.9	90%<br>10%	2314.1	85%<br>15%	1443.2
sp|Q8WXI9|P66B_HUMAN	13	K^{2}PPAPLLHFLPSA<br>K^{2}PPAPLLHFLPS^{1}A	2	50%<br>50%	341.2	56%<br>44%	339	83%<br>17%	223.7
sp|P55345|ANM2_HUMAN	13	PK^{2}Y^{1}NHILKPEDCL<br>PKY^{1}NHILK^{2}PEDCL	1	49%<br>51%	311	67%<br>33%	329.2	80%<br>20%	461.5
sp|Q8IXJ6|SIR2_HUMAN	14	Y^{1}R^{2}DVAWLGECDQGC<br>YR^{2}DVAWLGECDQGC	2	46%<br>54%	506	68%<br>32%	337.7	80%<br>20%	643.9
sp|Q96P70|IPO9_HUMAN	14	S^{1}VALCK^{2}LLQHGINA<br>SVALCK^{2}LLQHGINA	2	47%<br>53%	522.8	66%<br>34%	633.6	79%<br>21%	676.1
sp|Q9H9L4|KANL2_HUMAN	14	PDDLEAGPMDLY^{1}LS^1<br>PDDLEAGPMDLYLS^1	2	50%<br>50%	392.9	66%<br>34%	413.5	80%<br>20%	632.7
sp|Q9HCK8|CHD8_HUMAN	15	MR^{2}PDLS^{1}KMMALMQGG<br>MRPDLS^{1}K^{2}MMALMQGG	1	50%<br>50%	974.4	68%<br>32%	1422.1	78%<br>22%	1538.1
sp|P29375|KDM5A_HUMAN	15	NILPK^{2}R^{2}TRRVKTQSE<br>NILPKR^{2}TRR^{2}VKTQSE	1	52%<br>48%	566.3	68%<br>32%	707	80%<br>20%	573.1
sp|Q9NPF5|DMAP1_HUMAN	16	QK^{2}LIT^{1}AADTTAEQRRT<br>QK^{2}LITAADTT^{1}AEQRRT	1	50%<br>50%	656.1	68%<br>32%	442.7	80%<br>20%	429.4
sp|P25440|BRD2_HUMAN	17	DDIVLMAQT^{1}LEK^{2}IFLQK<br>DDIVLMAQT^{1}LEKIFLQK^2	1	50%<br>50%	613	68%<br>32%	689.4	80%<br>20%	503.1
sp|O94953|KDM4B_HUMAN	18	PPPPAHFPS^{1}EEALWLPS^{1}P<br>PPPPAHFPS^{1}EEALWLPSP	2	45%<br>55%	1533.2	68%<br>32%	1217.4	80%<br>20%	1277.1
sp|Q9UFC0|LRWD1_HUMAN	19	PK^{2}LEELS^{1}LEGNPFLTVNDN<br>PK^{2}LEELSLEGNPFLT^{1}VNDN	1	50%<br>50%	509	69%<br>31%	783.2	77%<br>23%	720.9
sp|Q9UNL4|ING4_HUMAN	20	R^{2}T^{1}EDLKAEIDKLATEYMSSA<br>RT^{1}EDLK^{2}AEIDKLATEYMSSA	1	53%<br>47%	409.2	68%<br>32%	464.2	79%<br>21%	366
sp|P23527|H2B1O_HUMAN	21	K^{2}AQK^{2}KDGKKRKRSRKESYSIY<br>KAQK^{2}KDGKKRK^{2}RSRKESYSIY	1	50%<br>50%	476.3	67%<br>33%	358.9	80%<br>20%	472

[1] Phosphorylation (UniMod ID: 21).
[2] Di-Methylation (UniMod ID: 36).

abundance of the proteoform A is 32% and the abundance of the proteoform B is 68%. Under the condition of this combinatorial abundance, these two proteoforms have the minimum sum of peak intensity errors. Besides, this spectrum can align 5 more peaks when identified as these two combinatorial proteoforms than when identified as a single proteoform.

For case 2, we give a detailed explanation for the case in the second row of Table S1, which is a

peptide with 8 amino acids from the protein $sp \mid Q96EZ8 \mid MCRS1\_HUMAN$. Fig. S4 shows the detailed graphical illustration of the quantification results.

As shown in Fig. S4, two proteoforms are identified and they are represented as two paths. For the proteoform A, the first PTM Phosphorylation occurs from the node pair $(41, 149)$ to the node pair $(51, 150)$ and the second PTM Phosphorylation occurs from the node pair $(73, 153)$ to the node pair $(89, 154)$. For the proteform B, there is only one PTM Phosphorylation occurred from the node pair $(71, 153)$ to the node pair $(85, 154)$. Since these two proteoforms have different total masses, the ending node pairs of these two proteoforms are different. In addition, the abundance of the proteoform A is 65% and the abundance of the proteoform B is also 35%. Under the condition of this combinatorial abundance, these two proteoforms have the minimum sum of peak intensity errors. Besides, this spectrum can align 5 more peaks when identified as these two combinatorial proteoforms than when identified as a single proteoform.

REFERENCES

1. Q. Kou, S. Wu, N. Tolić, *et al.*, "A mass graph-based approach for the identification of modified proteoforms using top-down tandem mass spectra," Bioinformatics **33**, 1309–1316 (2017).
2. Z. Zhan and L. Wang, "Fast peak error correction algorithms for proteoform identification using top-down tandem mass spectra," Bioinformatics **40**, btae149 (2024).
3. M. G. Awan and F. Saeed, "Mass-simulator: A highly configurable simulator for generating ms/ms datasets for benchmarking of proteomics algorithms," Proteomics **18**, 1800206 (2018).
4. X. Liu, S. Hengel, S. Wu, *et al.*, "Identification of ultramodified proteins using top-down tandem mass spectra," J. proteome research **12**, 5830–5838 (2013).
5. B. Ma, K. Zhang, C. Hendrie, *et al.*, "Peaks: powerful software for peptide de novo sequencing by tandem mass spectrometry," Rapid communications mass spectrometry **17**, 2337–2342 (2003).
